# Rationality Criteria for Complex Goal Systems: The case of the EU Water Framework Directive

**DOI:** 10.1007/s00267-026-02635-6

**Published:** 2026-10-10

**Authors:** Neelke Doorn, Karin Edvardsson Björnberg, Sven Ove Hansson

**Affiliations:** 1 Delft University of Technology, School of Technology, Policy and Management, Department of Values, Technology and Innovation, Delft, The Netherlands; 2 KTH Royal Institute of Technology, School of Architecture and the Built Environment, Stockholm, Sweden

**Keywords:** Water quality, Goal-setting, Rationality, Water Framework Directive

## Abstract

We extend a previous list of four rationality criteria for the setting of a single goal to a list of six rationality criteria for the construction of large goal systems, such as those commonly established for sustainability policies. These criteria are applied to the European Union’s Water Framework Directive (WFD). For each of the six criteria we find deficiencies that should preferably be redressed in future revisions of the framework. Based on this example, we argue that in the future, complex goal systems should be carefully analysed from the viewpoint of goal-setting rationality before they are adopted.

## Introduction

In light of climate change and environmental pollution, policies are needed to maintain the habitability of our planet, both now and in the future. Given the diverging stakes and the uncertainties surrounding climate change and sustainability more generally, research on the interface of sustainability and decision-making has expanded rapidly (Köhler et al. 2019), and the topic has been approached from various fields and disciplines. A burgeoning field, drawing on modeling techniques and computational approaches, is dedicated to decision-making under various types of uncertainty (Hansson 2018). Within the realm of policy sciences, scholars are examining the quality of decision-making processes related to climate change, emphasizing the importance of democratic participation and the inclusion of minority voices (Newig et al. 2023). Another body of literature addresses criteria for evaluating the overall quality of decision outcomes, emphasizing factors such as consistency, completeness, and practicality (Murphy and Gardoni 2007). It is worth noting that these discussions often intersect. What unites these diverse perspectives is the recognition that there are no universally applicable technocratic “best solutions.” Ultimately, most climate-related decisions carry political implications and should be made within the appropriate political forums, whether at the local, national, or international level.

There is an extensive discourse on the quality of policies, decision-making processes, and societal participation. However, comparatively little attention has been paid to the formulation of the corresponding policy goals. While goal-setting is almost universally recognized as a valuable tool in global environmental governance, there is limited understanding of how such goals influence the decisions and priorities of major stakeholders (e.g., national governments, authorities, and the private sector) and the outcomes that follow (Fukuda-Parr et al. 2014, Kanie et al. 2017; Young 2017). A growing body of literature has examined how to design policy goals to maximize the utility and effectiveness of goal-setting. For example, studies have examined how the clarity and ambiguity of climate goals affect their institutionalization and the mobilization of actors (Hofstad et al. 2021), as well as other characteristics that influence the effectiveness of goal-setting (e.g., Bjerke and Renger 2017; Edvardsson Björnberg, 2013; Fast and Widerberg 2025; Hilson 2020). However, few studies have addressed the challenges that arise in goal systems involving numerous goals and multiple agents and decision-makers who need to contribute to implementation. This research gap is particularly troublesome for policies with long-term aspirations, such as sustainability goals. Sustainability goals are typically multi-dimensional. They have long-term aspirations that primarily impact future generations who lack representation in the political arena. For such goals there is always a risk that short-term gains may overshadow long-term ambitions. Moreover, sustainability goals often face problems of spatial fit; that is, the geographical boundaries of the biophysical systems under consideration may not align with the mandates or areas of competence of the political and administrative institutions involved (Moss 2012). To date, the challenges associated with these complexities have received only limited attention in the literature on goal-setting. The present paper extends a previously proposed framework for the analysis of rational goal-setting to make it applicable to such complex goal systems.

We will use the European Water Framework Directive (WFD) as an example. We have chosen it since it involves to a high degree many of the complexities that make goal-setting in large sustainability issues difficult and controversial. In particular, its goals have to be designed to address the challenges associated with multiple overarching objectives, long time frames and lack of spatial fit. The WFD seeks to ensure both the qualitative and the quantitative health of water bodies by reducing and eliminating pollution while simultaneously supporting wildlife and meeting human needs. The WFD is an amalgamation of several former directives with differing subgoal formulations, which makes it a good illustration of some of the difficulties of combining different types of goals in a unified goal system. It also has the advantage of having given rise to an extensive secondary literature, in which the realism and adequacy of the goal system have been commented on.

The paper is structured as follows. Extending a previously proposed framework for the analysis of rational goal-setting, Section 2 introduces six rationality criteria for goals: precision, evaluability, approachability, motivity, completeness, and instrumental flexibility. Section 3 presents the Water Framework Directive and explains how its specific goals are formulated, after which Section 4 applies the six criteria to the Directive. This analysis identifies gaps in the directive and, in doing so, offers insights into the effectiveness of complex goal systems in environmental policy. Section 5 summarizes the findings and concludes.

## Rationality Criteria for Goal-setting

By a goal is meant a state of affairs that some agent intends to achieve or approach. Goals can stand in instrumental relations to actions, activities, and refrainment from action, as well as to other goals. If one goal is pursued in order to achieve or approach another goal, then the first goal is *instrumental to* the second. If an action, activity, or refrainment is performed in order to achieve some goal, then it is instrumental to that goal. It can also be said to be a *means* for the goal, and this type of instrumentality is often described as a *means-ends* relationship. Importantly, there is no clear boundary between means and ends (goals). A goal can simultaneously be pursued as an end in itself and as a means to achieve some other goal. We also often pursue goals without having decided whether they are ultimate goals or merely means to achieve some other goal. The distinction between means and goals can also shift between contexts. For instance, in environmental policy, the preservation of biodiversity is typically treated as an end in itself, but in a philosophical discussion it may well be asked whether the fulfillment of this goal serves as a means to achieving some other goal.

It is often assumed that subgoals created by a subdivision of a larger goal stand in a purely instrumental relationship to the more overarching goal. For instance, Ralph Keeney describes how a large goal or “fundamental objective” can be divided into parts, each of which specifies “means contributing to the achievement of any aspect of the fundamental objective” (Keeney, 1992, p. 16). He calls such goals “means objectives” and maintains that they are “only means to your ends” (ibid., p. 28). Their relevance in a decision-making situation derives from “the degree to which another (more fundamental) objective can be achieved” (ibid., p. 34). It follows that the subgoals (“means objectives”) add no new information on what has to be achieved. “[A] listing of only the fundamental objectives is appropriate to evaluate alternatives” (Gregory and Keeney 1994, p. 1040).

However, as far as we can see, such a purely instrumental (means-end) relationship is not a suitable ideal for the relationship between subgoals and overarching goals in large goal systems such as the WFD. A major reason for this is that the way in which an overarching goal is subdivided and responsibilized can be crucially important for the functionality of the goal system. For example, Wuijts et al. (2021) show that different water-quality objectives (hydrological, morphological and chemical) create different governance requirements, while more recent work on WFD implementation finds that achieving water-quality ambitions requires cross-sectoral choices involving agriculture, spatial development, and other policy domains (Wuijts et al. 2023). The authors find that stakeholders have different views on ambitions, achievements, and necessary actions, meaning that the overarching water-quality objectives do not straightforwardly determine what needs to be done by each sector. Therefore, the setting of subgoals will have to include an operationalization that specifies the reduction to take place in each of the sectors. This means that the subgoals are not purely instrumental but also express value-based prioritizations that do not follow from the overarching goal.

Goals are typically set by individuals or organizations (“agents”) to promote progress towards the ends they represent (Kanie et al. 2017). Thus, goal-setting aims to guide behavior by directing attention and resources, establishing priorities, motivating agents, and providing benchmarks against which progress can be evaluated (Young 2017). Edvardsson and Hansson (2005) use the term “achievement-inducing” to describe goals that contribute to their own achievement; that is, goals that are effective (functional, or rational). By rationality criteria for goals, we mean properties that goals should possess in order to successfully guide actions toward the corresponding end states. In the management literature, such criteria are commonly captured by the SMART acronym, which holds that goals should be specific, measurable, assignable, realistic, and time-related (Doran 1981).^1^ Edvardsson and Hansson (2005) propose four rationality criteria for individual goals: precision, evaluability, approachability, and motivity.^2^ However, some of the problems that arise in complex goal systems, such as those that have been developed for overarching sustainability policy, are not satisfactorily addressed by previously proposed criteria for goal rationality. We therefore propose additional aspects to be covered in the four criteria just mentioned, and we introduce two additional rationality criteria tailored to complex goal systems: completeness and instrumental flexibility.

What constitutes part of a goal system, and what lies outside it, is not always straightforward to determine. Complex environmental goal systems often comprise not only a set of environmental objectives, but also various legal and administrative arrangements designed to support their achievement. For example, in addition to the environmental objectives set out in Article 4 and Annex V, the WFD establishes reporting requirements (Article 15) and a system of penalties for breaches of national provisions adopted pursuant to the Directive (Article 23). These arrangements contribute to the fulfillment of the Directive’s environmental objectives and are therefore instrumentally valuable. Nevertheless, they do not form part of the Directive’s primary aim, namely, to achieve good status for all EU waters. The rationality criteria developed in this article apply primarily to the framework’s environmental objectives, rather than to the legal and administrative arrangements that support their achievement.^3^ However, it should be recognized that factors outside the environmental objectives themselves can affect how well the objectives contribute to their own achievement. That the person or body responsible for achieving an environmental objective participates in the goal-setting process is for instance often regarded as contributing to the goal’s functionality.

### Precision

A goal can guide action effectively only when the implementing agents know what the desired state described by the goal is and to what extent potential actions bring them closer to it. This requires that the goal is both precise and evaluable. Precision is the starting point, since evaluability presupposes that the desired state described by the goal is reasonably well specified. We turn to evaluability in the next section. Goals can vary in their degree of precision (Edvardsson and Hansson 2005). The most basic requirements of precision are that a goal should specify the direction in which agents must act to move closer to it (directional precision) and define a point in time by which it should be achieved or at least approached (temporal precision).

The degree of precision required for a goal to guide action typically depends on the social context in which it operates and the implementing agents’ background knowledge. Public policy goals such as those related to sustainability, are often deliberately ambiguous, frequently as a result of political compromise (Carrigan 2018). Politically, it may be more expedient to propose a vague goal capable of fostering unity and to specify details later, once a reasonable degree of consensus regarding the issue’s importance has been achieved. On the other hand, if a goal system refers to actions that must be performed by many agents with diverse knowledge and aspirations, a higher degree of precision may be required to ensure joint strivings in the intended direction. Sometimes, it is more efficient to adopt goals with directional precision first and to specify achievement levels and time frames later, once more information about feasibility constraints becomes available (Bjerke and Renger 2017). It is also often necessary to combine precision with flexibility concerning the means used to achieve the goals, see further Section “Instrumental flexibility”.

### Evaluability

While precision concerns the desired end itself, evaluability relates to the agent’s actions and their effects on goal achievement. A basic assumption in goal-setting theory is that goals regulate performance more effectively when actions are evaluated, and information about goal achievement is available to the responsible agents. Agents who know how far they have come in reaching the goal can more easily adjust their actions in the direction of the goal. Sometimes, information about the degree of goal achievement can be used to revise the goal itself. It is often difficult to determine in advance whether a certain policy goal is reasonably ambitious. This is especially true for many sustainability goals, for which there may be limited knowledge about how they play out in different social and geographical contexts. In such cases, information on what has been achieved relative to the goal can be used to recalibrate expectations about what can reasonably be achieved, thereby fostering motivation and sustained commitment to the goal.

Moreover, evaluability is a prerequisite for establishing accountability for insufficient goal achievement. This is particularly important in complex goal systems involving many decision-makers who report to one another and may be involved in various relationships of accountability. In such goal systems, evaluability refers not only to the achievement of the high-level goals of the system but also to the degree to which actions falling within the responsibility of various agents have contributed to goal fulfillment.

### Approachability

The third criterion is approachability. Approachability is closely related to the more commonly used criterion of achievability, but we argue that the former is more appropriate for our purposes. In goal-setting literature, it is often argued that goals should be challenging, yet possible to achieve given existing resources. Achievability is also one of the SMART criteria. A common argument for requiring achievability is that goals that are perceived to be unachievable risk becoming less motivating: As the agent realises that the goal will most likely not be achieved, their commitment may taper off. However, experience shows that challenging goals can often take us further in the desired direction than goals known to be fully achievable. The experience of the Vision Zero goal for road safety confirms this (Edvardsson Björnberg et al. 2023). The goal of zero deaths and serious injuries in road traffic is unrealistic in the sense that it can never be fully achieved. Nevertheless, it has served well to promote innovative solutions for traffic safety and to reduce the rates of severe injuries, beyond what would have been accomplished with goals adjusted to what was previously known to be possible. Moreover, trying to figure out what constitutes a realistic and perhaps even economically “optimal” target level in terms of the number of killed and seriously injured people does not seem like a morally acceptable approach (Rosencrantz et al. 2007). Essentially the same argument speaks against settling for “realistic” sustainability goals that are insufficient to ensure the attainment of a sustainable future within the planetary limits (Hilson 2020). At the same time, overly conservative goals may fail to induce the transformations required to address long-term environmental challenges, whereas ambitious environmental goals can provide a basis for legitimizing bold policy choices (Misonne 2015). Ambitious goals may play an important role in mobilizing action and innovation even when their full achievement is unlikely (Haarstad 2019). These considerations suggest that the relevant criterion is not whether a goal is fully achievable, but whether it can guide movement towards the desired state. We therefore use the term approachability rather than achievability in our analysis of the WFD.

While achievability is often viewed as a binary criterion, categorizing goals as either achievable or not, it is more appropriate to conceive of approachability as a matter of degree. A goal is more (or more easily) approachable if it can be reached with limited cost and effort, rather than requiring substantial sacrifice. Our weaker criterion of approachability must therefore be constructed as a graded concept to capture important nuances in goal fulfillment. This is particularly relevant for complex goal systems such as the WFD. In such composite systems, approachability must be assessed both for individual goals and for the system as a whole. Actions that advance one subgoal may either facilitate or hinder the achievement of others. Goal systems should thus be designed to minimize conflicts and promote synergies wherever possible (Nilsson et al. 2016). In some cases, this may require prioritization or compromise already at the goal-setting stage. To do so, we must (1) distinguish between the approachability of individual goals and the aggregated approachability of the goal system, (2) recognize different levels of approachability (or partial attainability) depending on how much of a goal can be reached, (3) account for the time frame within which that level of realization is achievable, and (4) determine the resources, efforts, and sacrifices required to achieve it within the specified time frame.

### Motivity

As noted above, a goal can be achievement-inducing not only through its action-guiding capacity but also by motivating progress towards its realization. However, combining motivity with precision and evaluability in a single goal can be challenging, since the latter two criteria often require technical specifications. Finnemore and Jurkovich (2020) argue that “lofty” goals, defined as broad, ambitious and often unattainable aims, are more effective in fostering consensus and encouraging actors to commit and take action (see also Fast and Widerberg 2025). In multi-level goal systems, this tension can often be resolved through a combination of motivating overarching goals and more precise and evaluable subgoals.

When achieving a goal requires contributions from many different agents, motivity depends on the alignment of their individual motives. Commitment to overarching goals alone may not suffice; agents must also be motivated to perform the tasks required for their part of the collective effort. This is well illustrated by many agents’ attitudes toward climate change: while recognizing the need for far-reaching mitigation measures, many justify inaction by demanding prior action from others (Birchall and Kehler 2023). In such cases, additional mechanisms may be needed within the goal system to motivate effective contributions, including informational, compensatory, or even punitive measures. Although these mechanisms are not part of the goal system itself and will not be discussed further here, an efficient goal system should be designed to maximize motivation and provide strong incentives for action.

Goal conflicts are often unavoidable in complex goal systems. If conflicts between goals induce or strengthen conflicts between persons, they can undermine motivity and thereby threaten goal achievement. This can easily happen if the fulfillment of each subgoal is delegated to a group that has responsibility only for that goal. It is usually much better to ensure that all groups share responsibility for advancing the goal system as a whole.

### Completeness

In sustainability and many other policy areas, overarching goals on higher political or administrative levels are often complemented by subgoals intended to support achievement of the overarching goals. The construction of such subgoals involves at least two processes:

#### Subdivision

Large goals are divided into smaller parts. The subgoals can be intended to be achieved sequentially, in parallel, or through some combination of sequential and parallel fulfillment.

#### Operationalization

Goals or subgoals contain additional information about what should be achieved, usually in order to enhance precision and evaluability.

The creation of subgoals is often combined with responsibilization. By this is meant that agents are made responsible for (sub)goals or for actions that will contribute to achieving subgoals. Responsibilization is not a constitutive part of the goal system per se, and will not be further treated here.

It is commonly assumed that goals and subgoals should be aligned such that the highest-level goal is achieved or at least approached to a significant degree if all subgoals are reached. We may refer to this property as completeness. Completeness requires that the targets, taken together, comprehensively reflect the most salient aspects of the overarching goal. Thus, adequate subdivision and/or operationalization of an overarching goal has to be based on adequate and detailed knowledge of what is required to achieve or meaningfully approach the goal.

It could also be required that the subgoals of a goal have no overlap, so that no requirement follows from more than one of the subgoals. It would seem to be an advantage to avoid the risk of double-counting that follows from overlaps. However, in practice there are many actions that have to be taken for several reasons. For instance, it is sensible to have both a goal requiring that the water in some surface water is suitable as a source for drinking water production, i.e. non-toxic to humans, and a goal requiring that it is non-toxic to aquatic fauna. There is a considerable overlap between these two goals, for instance they both require that PFAS concentrations are as low as possible. This overlap reflects the fact that we have two important reasons to avoid these contaminants. Other overlaps may be less desirable, but the fact that some overlaps are appropriate is reason enough not to introduce a requirement that overlaps should be avoided.

### Instrumental Flexibility

If an operationalized goal or subgoal specifies the use of a particular method or technology, it can become obsolete when new and better technological solutions become available. This is why environmental regulation often requires the use of the “best available technology” (BAT) rather than a specific technology, and why regulation of ionizing radiation exposure requires exposures to be “as low as reasonably achievable” (Hansson 2023). In general, rational goal operationalizations should allow and encourage the use of the best available methods for goal achievement. Theoretically, this is achieved by ensuring that the goal system specifies only ends, not the means to achieve them. In practice, however, the means-ends distinction is often blurred. To be action-guiding, a goal system must often include goals whose realization serves as a means to achieving other goals. For instance, if the overarching goal is safe drinking water, a subgoal may specify a maximum concentration of some toxic substance. Yet, specifying the technical method for reaching this concentration may render the subgoal obsolete when a better method becomes available. Thus, an appropriate level of flexibility in the goal system cannot be defined solely by the means-ends distinction. Instead, it must balance two opposing risks that can both reduce goal-achievement efficiency: excessive leeway for implementing agents, which may weaken contributions to goal attainment (e.g., if “toxic levels” are left undefined), and overly detailed prescriptions, which may prevent the adoption of improved methods (e.g., specifying a particular purification process). Instrumental flexibility is therefore essential to balance these risks and maintain the goal system’s achievement-inducing capacity.

Another way to achieve flexibility is to allow for expeditious revision of the goal system, in particular if new knowledge makes some subgoal inefficient. This is a solution outside of the goal system, per se. It will not be treated further here.

## The Water Framework Directive

Adopted in 2000, the European Water Framework Directive (WFD) is the EU’s main law for protecting and improving water resources (Voulvoulis et al., 2017). It replaced a fragmented set of earlier directives, each addressing narrow aspects of water quality, and created an integrated framework covering surface waters, coastal waters, transitional waters, and groundwater. The WFD broadened water quality assessment beyond chemical pollution to include ecological status, marking a shift in EU water policy. While the determination of water quality in the previous century primarily focused on the presence of toxic substances, the WFD also encompasses an ecological assessment of water, which goes beyond limitations of the concentrations of specified chemical substances. Its objectives are to achieve “good status” for all water bodies, prevent deterioration, and promote sustainable use. Due to its integration of existing laws and its ambitious scope, the WFD is complex, with differing definitions of good status for surface waters, subdivided into rivers, lakes, transitional waters, and coastal waters, and for groundwater.^4^ While the structure for different water bodies is more or less the same, the exact criteria differ in detail. In this paper, we focus on the WFD as it applies to surface waters, and we take the WFD as it applies to rivers to illustrate its goals.

*Set-up of the Water Framework Directive*

The quality of the surface waters is assessed based on two overall criteria: the ecological status and the chemical status, each consisting of various subcriteria. The status of a water body is the general expression of the status of that water body, determined by the poorer of its ecological status and its chemical status (Article 2, sub 17), where the status is classified, in order of improving quality, as bad, poor, moderate, good, or high. Hence, achieving “good status” means that both the ecological status and the chemical status of a water body are at least good (Article 2, sub 18).

*Ecological status*

The ecological status includes a combination of biological elements (e.g., species richness of animals and plants), chemical, and physico-chemical quality elements (e.g., phosphorus and nitrogen levels, water temperature, presence of so-called priority substances designated by the EU as hazardous to human health or the environment and requiring priority action), and hydromorphological elements, referring to the “shape” of the water bodies, such as the degree of meandering, bank steepness, erosion levels, and the ease of organism movement. The hydromorphological characteristics are crucial for the ecological functioning of the water body: they determine whether specific organisms can inhabit the water. The WFD specifies the elements to be considered in determining the ecological status. However, it does not prescribe for all elements *how* they should be determined or what the respective values should be. This ultimately grants member states considerable freedom in operationalizing the ecological status of water.

*Chemical status*

The second criterion is the chemical status of the water, which is defined in terms of the maximum allowable concentrations of specific pollutants. These concentrations are set at the European level, so member states do not have the freedom to establish higher maximum concentrations themselves.

*Classification*

Based on these two criteria, all waters are classified on a 5-point scale ranging from “high status”, via “good status”, “moderate status”, and “poor status” to “bad status”. In order to comply with the WFD, all designated waters should be classified “good status” by 2027, which means that both the ecological status and the chemical status of a water body are at least good (Article 2, sub 18). For the ecological status, only the biological quality elements need to be fully specified to classify them into one of the five status categories. The values of the hydromorphological quality elements must only be taken into account when assigning water bodies to the high ecological status class, and for the values of the physico-chemical quality elements only when assigning water bodies to the high and good ecological status classes. For the other status classes the hydromorphological and physico-chemical elements are expected to have “conditions consistent with the achievement of the values specified for the biological quality elements” (WFD Annex V 1.2.1).

### Specification of the Goals

#### Ecological Status

The WFD requires member states to establish the type-specific biological, hydromorphological, and physico-chemical reference conditions representing values associated with high ecological status for the relevant quality elements (Annex II 1.3, (i)).

The biological quality elements are expressed as unit-less ecological quality ratios or fractions of reference values set by the member states. The use of ratios ensures the comparability of results between member states. The ratio is expressed as a numerical value between zero (worst class) and one (best class). The directive provides that member states may use species or groups of species representative of the quality element as a whole in their monitoring systems (WFD Annex V 1.4.1, sub (i)).

The physico-chemical conditions are expressed as ranges that should not be exceeded. The ranges or levels that member states establish for the general physico-chemical quality elements should be as ecologically relevant as current expert knowledge permits (EC, 2003a, p. 15). For nutrients, the WFD does not refer to type-specific values but to ecosystem functioning generally (WFD Annex V 1.2.1). The concentrations of specific pollutants should not exceed environmental quality standards set in accordance with WFD Annex V 1.2.6.

For the hydromorphological elements, only the conditions associated with high ecological status need to be specified. In the WFD, these are defined as those under which “there are no, or only very minor, anthropogenic alterations to the values of the (…) hydromorphological quality elements for the surface water body type from those normally associated with that type under undisturbed conditions” (WFD Annex 1.2). This means that the quantity and dynamics of flow, and the resultant connection to groundwaters, are the same as under totally, or nearly totally, undisturbed conditions (hydrological regime).

#### Chemical Status

Contrary to the different elements under the ecological status, the classification of the waters’ chemical status is uniform across all Member States. “Good surface water chemical status” requires that the concentrations of a prescribed list of pollutants do not exceed the environmental quality standards established in the WFD and other relevant Community legislation (WFD, Article 2, sub 24).

Table 1 presents a summary of the specification of the evaluation of the ecological status and chemical status.

**Table 1:** Specification of the evaluation of ecological status and chemical status criteria

Criterion	Unit	Discretion
**Ecological status**		
− Biological	Ratio	Relevant elements and their thresholds selected for each water body by the member state
− Physical-chemical	Absolute values/ concentrations	− General elements: elements prescribed at EU level, specific thresholds specified per ecosystem type by the member state − Nutrients: elements prescribed at EU level, thresholds specified by the member state − Pollutants: both elements and thresholds prescribed at EU level
− Hydromorphological	Pass/fail	Elements prescribed at EU level, specific thresholds specified per ecosystem type by the member state
**Chemical status**	Concentrations	Both elements and thresholds prescribed at EU level

## Applying the Rationality Criteria to the WFD

### Precision

As argued above, a basic requirement of precision is that a goal should indicate the direction in which agents must act to approach it. This requirement is largely satisfied by the WFD, both in its overall purpose (Article 1) and environmental objectives (Article 4), and in the quality elements it identifies (Annex V). However, the directive provides much less guidance on the degree to which the desired states should be achieved at specific points in time. One potential source of unclarity is the normative definitions of ecological status classifications stated in Annex V. The directive identifies *high status* in terms of “no, or only *very minor*, anthropogenic alterations to the values of the physico-chemical and hydromorphological quality elements […] from those *normally* associated with that type under undisturbed conditions”, and *good status* in terms of only slight such deviations; however, with no further direction as to how “very minor” or “slight” should be interpreted (Moss 2008). These terms are obviously vague. More problematic still is the reliance on *reference conditions*—the benchmark for assessing anthropogenic alternations—which is based on the widely criticized idea of stable and predictable ecological states. Ecologists have long pointed out that ecosystems are inherently dynamic in terms of species composition and population sizes, and that notions such as “undisturbed conditions” are of limited utility as policy guides in the Anthropocene (Bouleau and Pont 2015).

Nature preservation aims to avoid the effects of human activities. Nature restoration aims to remove the effects of human activities that have already taken place. Although the WFD does not refer to naturalness, it specifies the highest status of water as the conditions normally associated with “undisturbed conditions” (WFD Annex V, Article 1.2, Table 1.2). For chemical substances it proclaims “the ultimate goal of achieving concentrations in the marine environment near background values for naturally occurring substances and close to zero for man-made synthetic substances” (WFD Article 1). These statements indicate that nature should be restored to how it was before any human interference.

However, goal setting for preservation and restoration of nature is more complex than that. Although conservationists and nature restoration practitioners usually strive to avoid and remove the effects of human activities on nature, they do not always do so. In some cases, they accept and even strive to preserve effects of human activities. The biodiversity that has developed over centuries in cultural landscapes, for instance on meadows and in ditch banks, is usually considered to constitute biotopes that should be preserved, rather than restored to some pre-agricultural state. Nature conservation in areas with a long history of human impact will therefore always have to be based on some kind of delimitation between those human impacts on nature that should be preserved and those that should be reversed as far as possible (Phillips 1998). This applies to a large extent to European waters, since they have undergone millennia of transformation through the construction of bridges, dams, canals, and other interventions related to water power, shipping, fishing, among others. One could therefore expect the WFD to provide guidance on this delimitation, yet it does not.

### Evaluability

For the criterion of evaluability, a distinction needs to be made between on the one hand the evaluability of the different elements (subgoals) that comprise the goals of good chemical status and good ecological status respectively, and on the other hand the evaluability of the overall goal system. To increase accountability, the WFD formulates the elements within the goals of good chemical status and good ecological status in such a way that progress towards goal achievement can be compared between member states.

For the chemical status, the subgoals are specified in terms of measurable threshold concentrations. They provide clear information on where the agent stands in relation to the specific subgoal. The threshold concentrations are set at the European level, which allows comparability between member states. This goal is in principle readily evaluable.

For ecological status, the WFD requires member states to establish type-specific biological, hydromorphological, and physico-chemical reference conditions representing values associated with high ecological status for the relevant quality elements (Annex II 1.3(i)). Biological quality elements are expressed as unitless ecological quality ratios or fractions of reference values set by member states to enable comparability between them. Physico-chemical quality elements are defined in absolute values or concentrations, making progress on these elements evaluable. Hydromorphological elements occupy a different position within the goal system: only the conditions associated with high ecological status must be specified (see Section “The Water Framework Directive”), formulated as totally or nearly undisturbed conditions. Examples of disturbed conditions include “altered flow regime” or “altered sediment dynamics”. This results in a binary evaluation, making it difficult to assess intermediate progress toward goal achievement.

With the exception of the hydromorphological elements, the different elements within the two overall goals of good chemical status and good ecological status provide in principle action-guiding information on goal achievement. However, the overall goal system obscures some of this information.

Goal fulfillment is based on the “one-out, all out” (OOAO) principle, which means that the overall status of a water body is determined by its lowest-scoring elements. Even if most elements meet the high status standard, a single failing element results in the entire water body being classified at the lower status level. While introduced to ensure that all aspects of water quality are addressed, preventing partial improvements from masking critical issues, the OOAO principle has also been criticized for making it difficult to show gradual progress. Hering et al. (2010) observe that the principle may increase the likelihood of type I errors; that is, mistakenly concluding that a water body is below good status even when it actually meets this criterion. This, in turn, could invoke unnecessary costs, since measures might be taken where they are not really needed (Borja and Rodríguez 2010).

Hering et al. (2010:4013) criticize the OOAO principle also on the grounds that “it contrasts with the ecosystem approach the WFD is pursuing, as it is scientifically difficult to justify that a single component determines the quality of an ecosystem”, which makes the OOAO principle also problematic from the point of view of completeness.

A system of evaluation can only fulfill its purpose if the outcome of an evaluation reflects the degree of goal-achievement. If a water system that is far from overall goal achievement can receive the same assessment as one that is close to achieving the overall goal, then the evaluation system is misleading and cannot be used for guidance. Therefore, the OOAO principle needs to be replaced by a more adequate method for aggregating measures of subgoal achievement.

Another aspect of evaluability concerns the directive’s operationalization. The WFD goals are to be implemented locally through River Basin Management Plans (RBMPs), which translate the directive’s ambitions and legal mandate into action. The deliberate ambiguity of the overarching goals supports this process by allowing adaptation to local circumstances, considering both economic feasibility and uncertainties in water management. However, such flexibility must be balanced against the need for enforceable mechanisms. Vague or ambiguous goals may prevent weak implementation from being identified and corrected, ultimately undermining the directive’s aim of achieving good water status. Some scholars argue that the directive’s structural features, especially the broad discretion granted to member states and weak enforcement mechanisms, significantly compromise its ambitions (Green et al. 2013).

### Approachability

The WFD goals have been criticised for being insufficiently realistic. For instance, Hering et al. (2010) argue that in order to achieve good ecological status in all surface waters, rehabilitating actions need to be taken over many years, in some cases decades or even centuries. In the scholarly literature, there seems to be consensus that judging by the previous and present pace of implementation, the overarching goal of good water quality by 2027 is not achievable (Albiac et al. 2024). This also seems to be the opinion among practitioners engaged in implementing the directive. In recent survey, regional water authorities in the Netherlands considered it unlikely that the WFD objectives will be reached by 2027 (van Kats et al. 2022). However, this appears to depend primarily on delayed implementation and a lack of recognized urgency (Carvalho et al. 2019). Soon after the WFD entered into force, many countries signaled that they would not aim for compliance at its first evaluation, instead postponing the deadline to the latest permissible date (2027). Experience from climate policy and environmental management more broadly shows that delaying action compromises the effectiveness of measures (e.g., Naujokaitis-Lewis et al. 2018) or, at the very least, substantially increases the costs of effective action (Bosetti et al. 2009). Nevertheless, this should not be taken to mean that the goals themselves are unachievable (Karlsson and Gilek 2020).

For the weaker criterion of approachability, this varies across subgoals in the WFD goal system. A key distinction lies between point and non-point pollution, which are regulated differently. Point pollution has a clear causal link between an activity and the release of pollutants into surface water at a specific location, whereas such a link is harder to establish for diffuse or non-point pollution. The WFD therefore requires point-source activities to be regulated in advance (Article 11, sub 3(g)). Such pollution emanates mainly from urban and industrial sources. For both types of sources, substantial progress has been made, and further improvement using similar measures remains feasible (Albiac et al. 2024). Although full goal achievement by 2027 appears unlikely for point pollution, these goals remain broadly approachable.

Non-point pollution of water bodies comes primarily from agriculture. It consists mainly of nitrate that leads to eutrophication and pesticides that have ecotoxic effects. In both these cases, progress is small or non-existent. Nitrate pollution and eutrophication from fertilization are high and increasing in several major basins. (Albiac et al. 2024). A comprehensive study based on monitoring of agricultural pesticides in European water bodies showed considerable improvement in monitoring practices, but it also showed increased concentration of pesticides (Wolfram et al. 2021). Technologies are available for reducing emissions to water of both nitrate and pesticides. Both fertilizer and pesticide use can be considerably reduced without negative effects on harvests (Zanin et al. 2022). Vegetated canals and ditches can reduce the eutrophication induced by nitrate emissions (Soana et al. 2023). However, policy measures that ensure the implementation of these and other technologies that reduce water pollution from agriculture are not in place. One reason for this may be that the “polluter pays” principle that has proven efficient to reduce industrial pollution is less suitable for non-point pollution due to difficulties in determining the origin of pollutants (Albiac et al. 2024). We conclude that the goal of reducing non-point pollution from agriculture is approachable, but only if new regulatory tools for that purpose are introduced and used on a broad scale (Bieroza 2021).

The approachability of pollution reduction goals for both point and non-point sources comes with an important caveat: climate change will negatively affect Europe’s aquatic ecosystems. Flooding is expected to increase in the north, while droughts will become the main issue in the south. Rising surface water temperatures may further harm biodiversity. Consequently, many European water bodies, especially those not yet in good status, face heightened risks of degradation. For the WFD, this may, in some cases, “make the achievement of the objectives outright impossible” (Puharinen 2021, pp. 1-2). In addition, climate adaptation measures such as flood defense infrastructure can have negative effects on water quality objectives (Hendry 2017). It does not seem possible to balance out the negative effects of increased water temperature with any type of countermeasures. This means that the approachability of key water quality objectives is conditional on efficient measures to mitigate and adapt to climate change.

### Motivity

There are two fundamental ways in which goals, such as those articulated in the WFD, can contribute to intended policy outcomes. Goals—here, the achievement of good chemical and ecological status for European surface waters—together with the criteria for measuring progress, provide a foundation for planning and coordinating action across time and between agents. With reference to the WFD, decision makers can plan and allocate tasks to different agents, while ensuring that everyone understands what needs to be done, when it should be done, and (ideally) how it fits into broader initiatives at local and national levels (Wuijts et al. 2023). Moreover, the WFD goals serve as a motivational source, initiating and sustaining action over time and across diverse stakeholders. A central question, therefore, is whether the WFD goals are set in a way that induces and sustains the desired forms of action. Although few studies have, to the best of our knowledge, investigated the motivational capacity of the WFD from the perspectives of various stakeholders, several issues warrant further attention.

First, research in goal setting theory and practice has shown that the motivating capacity of a goal is tightly linked to the criteria of precision, evaluability, and approachability. Psychological studies indicate that goals that are precise, challenging, and accepted by the agent tend to elicit greater effort, both in terms of intensity and durability, than vague or do-your-best goals (Locke and Latham 2002). Similarly, studies suggest that goals that are reliably evaluated (and, thus, evaluable) tend to promote stronger efforts and improved performance (Locke and Latham 2019). This effect is partly believed to originate from the motivational impact such goals may have as agents are able to determine their current standing relative to the goals.

Most studies on the application of goal-setting theory focus on individuals or relatively small organizations, such as firms or workplaces. Caution is therefore required when extending the findings to public policy contexts. Nevertheless, if the findings can reasonably be extended to the WFD, this would imply that water managers, political decision-makers, water-related NGOs, farmers and agricultural companies, etc. are more likely to commit when they understand what they are aiming for; when what they are aiming for is within reach; and when progress can be measured reliably enough to build momentum. As noted above, the WFD goals only partially satisfy these requirements.

Another crucial factor for stimulating goal commitment is what is referred to as “ownership”, that is, the extent to which those responsible for implementation perceive the goal as relevant (MacLeod 2012). A goal that is considered relevant will more easily build commitment to sustain coordinated efforts towards goal achievement. What makes a goal relevant in this sense is of course largely its content, in the case of the WFD whether good chemical and ecological status of water bodies is regarded as desirable. But it may also be shaped by procedural factors, such as whether those responsible for implementing the goals have been included in the goal-setting process.

Article 14 of the WFD requires that member states engage with the people who live, work, and recreate in a catchment. Thus, stakeholder participation is built into the directive. However, because water management remains largely sectoral rather than cross-sectoral in many member states (Söderberg 2016), the directive’s ability to engage and motivate agents outside the water policy field is limited (Wuijts et al. 2023). Weak alignment with adjacent policy areas, such as agriculture, transportation, and spatial planning, undermines policy coherence, creates confusion, and could ultimately reduce commitment among key stakeholders.

Finally, the extensive use of exemptions and derogations, permitted under Articles 4.4-4.7 of the directive (see Section “Instrumental flexibility”), may further weaken the motivational capacity of the WFD goals. When such provisions are frequently used to justify delays or lowered ambitions, they risk generating skepticism about the seriousness of the commitment to real progress. Over time, this could contribute to aspiration fatigue; that is, when aspirational goals regularly go unmet this may foster cynicism or discouragement and, ultimately, reduce willingness to sustain efforts towards goal achievement (Finnemore and Jurkovich 2020).

### Completeness

Despite being an unusually comprehensive and wide-reaching regulation, the directive contains some remarkable gaps that need to be addressed with additional subgoals in order to satisfy the criterion of completeness. The following is a non-exhaustive list of issues that have been identified in the literature.

Climate change has been called the “elephant in the room” of the directive (Hendry 2017). Considering the effects of climate change on European water bodies should be explicitly considered in the directive. While in 2009, 2012, and again in 2015, the Commission noted that there was insufficient information about the impacts of climate change, the IPCC reports indicate that the impact of climate change for floods and drought will be huge, whilst both mitigation and adaptation to climate change will affect land use and water management.

The directive’s focus on water quality overlooks the growing challenge of water scarcity (Albiac et al. 2024). Moreover, it lacks goals to promote water-efficient technologies in industry and agriculture, which are both essential for the sustainable management of limited water resources (Rahaman et al. 2004). This omission is especially problematic for agricultural pollution, given the multiple ways in which drought and such pollution are interconnected. Drought reduces water availability, lowers agricultural productivity, and increases pollutant concentrations in remaining water sources. Conversely, intensive farming can cause pollution that further degrades water and soil quality, making ecosystems more vulnerable to drought.

The directive’s regulation of chemical pollution is entirely focused on specified substances that are known to have harmful effects on the environment or human or animal health. Unfortunately, there is considerable uncertainty about the potential effects of other substances that may contaminate European waters. For the vast majority of chemical substances in use, there is little or no information about toxicity or ecotoxicity (Rudén and Hansson 2010; Liang et al. 2025). In the WFD, substances with unknown properties are treated in the same way as substances that have been tested without any sign of toxic effects. This is inadequate, since the risk of unknown harmful effects is much larger for the untested chemicals. Several measures are needed to reduce the potential risk of toxic effects from untested substances in European waters. Efficient such measures can include: (1) a European program for identifying and measuring unknown contaminants in water, (2) a mechanism in European legislation for precautionary limitations of contaminants for which basic toxicity and ecotoxicity data are lacking, and (3) a system for prioritization of toxicological and ecotoxicological testing of such substances.

Contrary to most other chemicals, pesticides can only be used in Europe after a registration procedure that requires data on toxicity and ecotoxicity. However, many of the registered substances can have considerable ecotoxic effects at high exposure levels. Therefore, monitoring of pesticide concentration is essential. Unfortunately, too few pesticides are included in the obligatory monitoring programs; for example, in Germany, only 18 of the 301 pesticides approved for use are covered (Weisner et al. 2022). A goal requiring more comprehensive monitoring should be introduced.

Finally, 60% of the waters covered by the directive are transboundary. Although the directive provides a framework for collaboration between member states, its goal system offers no specific guidance for preventing or resolving water-related conflicts between them (Josefsson 2025). Given the shared nature of these resources, the absence of such guidance increases not only the risk of disputes, but also the risk of ineffective measures.

### Instrumental Flexibility

In principle, the WFD reflects the criterion of instrumental flexibility by setting environmental goals, such as achieving “good ecological and chemical status” for all water bodies, without prescribing the exact means to achieve them. In practice, however, the WFD’s flexibility is mainly operationalized through its system of exemptions, which both enable and constrain adaptive implementation.

Article 4 of the WFD outlines five categories of exemptions. The most commonly invoked is a deadline extension (Art. 4[4]), which allows member states to postpone the 2015 deadline to 2027 if objectives are technically infeasible, disproportionately costly, or unachievable due to natural conditions. While this clause introduces necessary temporal flexibility, it has also led to delays in implementation. Many member states anticipated missing the original targets and defaulted early on to the extended timeline. The “no deterioration” clause is intended to safeguard ambition but has not prevented significant lags in progress (Boeuf et al. 2016).

Less stringent environmental objectives (Art. 4[5]) introduce further flexibility, allowing lower targets where achieving good status is infeasible or too costly. However, justifications for such exemptions are often inconsistent. Methods for evaluating disproportionality vary widely—from cost-benefit analysis to ability-to-pay—and there is no universally accepted framework across the EU (Klauer et al. 2016). This lack of methodological clarity undermines the transparency and comparability of decisions.

Other exemptions accommodate external shocks or development needs. Temporary deterioration due to exceptional or unforeseeable events (Art. 4[6]) and new modifications in the public interest and/or beneficial to the environment or society (Art. 4[7]) allow deviations from objectives, provided that mitigation measures are applied and no less harmful alternatives exist. While these clauses allow for the necessary responsiveness to changing conditions, they also risk enabling environmentally detrimental projects under vague criteria (Boeuf et al. 2016).

The designation of Heavily Modified and Artificial Water Bodies (HMWB/AWB) (Art. 4[3]) is a structural form of flexibility. These water bodies are not required to reach “good ecological status” but must achieve the “best ecological potential,” given their uses (e.g., flood control, navigation). Crucially, this designation must be reviewed every six years, providing a formal opportunity to revise objectives in light of technological advances or changing socio-economic conditions (EC, 2003b).

Although most discourse on WFD flexibility focuses on justifying lower ambition, the goal system in principle allows for increasing stringency over time. For example, waters designated as heavily modified can be reclassified as natural water bodies if improvements in technology or infrastructure make restoration feasible, thereby reintroducing stricter ecological standards. Similarly, the list of priority substances can be updated and sharpened, allowing the regulatory scope to evolve. However, in practice, member states have often lobbied for more lenient measures rather than raising environmental ambitions, risking a gradual erosion of the directive’s original ambition.

Hence, while the WFD incorporates elements of instrumental flexibility through exemptions and periodic reviews, safeguards to preserve the original level of ambition of the WFD are largely lacking. Without stronger provisions for continuous improvement and harmonized justification standards, flexibility risks becoming a means of lowering ambition rather than adapting effectively to achieve environmental goals.

## Discussion and Conclusion

To investigate the goal-setting rationality of the Water Framework Directive (WFD), we applied a set of six rationality criteria. These criteria build on an earlier list of criteria developed for the evaluation of individual goals, with two new criteria added to address issues that arise in large and complex goal systems such as the WFD. Our analysis of the WFD in relation to these criteria revealed weaknesses across all six dimensions.

### Precision

One of the major difficulties in defining the aims of nature preservation is determining how, and to what extent, biodiversity created through long-term human interventions (“cultural landscapes”) should be protected. The WFD provides no guidance on this issue, but speaks about “undisturbed conditions” as if these were always well-defined. This ambiguity leaves considerable room for different interpretations, which makes the goals less precise than what they could have been.

### Evaluability

Under the WFD, overall goal fulfillment is evaluated according to the “one-out, all out” principle. This means that if two water bodies have an equally bad status in their respective weakest dimension, then they are taken to have the same overall status, even if one of them performs substantially better in all other dimensions. This principle makes it impossible to evaluate progress in water management in an acceptable way. It should be replaced by a more adequate method for aggregating subgoal achievement.

Furthermore, for hydromorphological goals, the WFD often only specifies desirable end states without providing any guidance for grading different degrees of partial satisfaction of the goals. This also makes it difficult to evaluate different degrees of progress towards the goals.

### Approachability

The extent to which the WFD goals are in fact approachable will depend largely on the effectiveness of future climate mitigation measures. Whereas the goals of the WFD would be approachable (and in many cases also attainable) in the absence of anthropogenic climate change, their actual degree of approachability depends on the future climate. In a realistic assessment of approachability, its dependence on climate mitigation needs to be clarified.

### Motivity

We have argued that the extensive availability of exemptions in the WFD that allow for lowered ambitions threaten the motivational capacity of the framework. We propose that a more restrictive approach to exemptions should be taken in future revisions of the framework. Improved policy alignment and more effective evaluation mechanisms are needed to ensure that all stakeholders involved in the WDF’s implementation share responsibility for the directive as a whole. Further research should examine the directive’s motivational capacity from the perspectives of different stakeholder groups.

### Completeness

We have identified several significant gaps in the WFD. The challenges posed by climate change, such as more frequent and severe droughts and flooding, are not addressed, nor is the risk of future water scarcity. The goals concerning chemical pollution cover only substances with known toxic effects, not the potential risks of substances with unknown properties. No guidance is given for resolving water-related transboundary issues, such as downstream impacts of increased water use. These and other gaps need to be addressed to ensure sustainable solutions to Europe’s water-related challenges.

### Instrumental Flexibility

The WFD’s flexibility primarily allows for lower ambitions. We propose replacing this with a consistently instrumental flexibility. This means retaining or, whenever possible, increasing flexibility in the choice of means to achieve the goals, while reducing flexibility in the goals themselves, i.e., in what to achieve.

We propose that these deficiencies in the WFD should be amended in future revisions.

Concerning the evaluation of complex goal systems, our analysis also highlights two qualifications concerning the six criteria. First, the criteria should not be understood as fully independent dimensions. There are important interdependencies between them: for example, evaluability depends partly on precision and completeness, while motivity may depend on both evaluability and approachability. As also indicated in our description of the criterion of completeness, we do not consider the absence of overlap between subgoals a necessary requirement for a goal system, and the same applies to our overall criteria. Consequently, some shortcomings may also reasonably be identified under more than one criterion. The purpose of distinguishing the criteria is therefore not to establish mutually exclusive categories, but to provide analytically distinct perspectives for assessing different properties of a goal system. A more systematic analysis of the relationships between the criteria may help identify their core components and clarify where their analytical boundaries lie. Second, we indicated that the arrangements required to implement a goal should be distinguished from the goal system itself. Institutional responsibilities, coordination mechanisms, resources, and other implementation arrangements are not properties of the goals, but they are instrumental to achieving them. Assessing the adequacy of these arrangements therefore constitutes a complementary, rather than a constitutive, part of evaluating a goal system.

Taken together, our analysis demonstrates the value of examining goal systems systematically before they are adopted. By making different dimensions of the rationality of the goal system explicit, the six criteria provide a structured basis for identifying strengths, weaknesses, and potential tensions within complex goal systems. The methodology introduced in this article can thus support more deliberate and transparent goal-setting, helping policymakers and other actors to assess whether proposed goals provide a sufficiently sound basis for guiding action towards desired future states.

## Data Availability

No datasets were generated or analysed during the current study.

## Footnotes

1 There is considerable variety in what the SMART acronym stands for (Wade 2009). For instance, the letter S has been associated with the words specific, significant, stretching, simple, strategic, and sensible, and the letter A with the words assignable, achievable, attainable, agreed, and accessible, among others.

2 There is significant overlap between the SMART criteria of specificity, time-boundedness, and measurability with Edvardsson’s and Hansson’s (2005) rationality criteria of precision and evaluability. However, the SMART criteria do not include any criterion that straightforwardly corresponds to their criterion of motivity. The criteria of achievability and approachability are not entirely overlapping, as explained in section “Approachability”.

3 We are grateful to one of the reviewers for drawing our attention to this point.

4 The WFD distinguishes between “ordinary” surface waters (rivers, lakes, transitional waters, coastal waters) and artificial and heavily modified surface waters. Ordinary surface waters must meet all subcriteria: both the ecological status and the chemical status must be good. Less stringent requirements apply to artificial and heavily modified surface waters. These waters need to have sufficient ecological potential, which is less stringent than achieving a good ecological status
